# Utility of a Novel High‐Sensitivity Multiplex Companion Diagnostic Test Using Formalin‐Fixed Paraffin‐Embedded Cell Block Materials of Non‐small Cell Lung Cancer

**DOI:** 10.1002/cam4.71028

**Published:** 2025-07-04

**Authors:** Yoshiki Shinomiya, Yuko Ishida, Kanako C. Hatanaka, Mayumi Yamamoto, Ayae Nange, Asami Okumura, Yumi Wada, Mitsuharu Abiko, Tomohiro Shimizu, Ryoko Watanabe, Yamato Hashimoto, Yoshiharu Sato, Jun Sakakibara‐Konishi, Tatsuya Kato, Shinya Tanaka, Yutaka Hatanaka

**Affiliations:** ^1^ Center for Development of Advanced Diagnostics, Hokkaido University Hospital Sapporo Japan; ^2^ Department of Surgical Pathology Hokkaido University Hospital Sapporo Japan; ^3^ DNA Chip Research Inc. Kawasaki Japan; ^4^ Department of Respiratory Medicine Faculty of Medicine, Hokkaido University Sapporo Japan; ^5^ Medical Network and Welfare Center, Hokkaido University Hospital Sapporo Japan; ^6^ Department of Thoracic Surgery Hokkaido University Graduate School of Medicine Sapporo Japan; ^7^ Department of Cancer Pathology Faculty of Medicine, Hokkaido University Sapporo Japan; ^8^ Institute for Chemical Reaction Design and Discovery (WPI‐ICReDD), Hokkaido University Sapporo Japan

**Keywords:** cytology, EGFR, next‐generation sequencing, non‐small cell lung cancer, tumor content

## Abstract

**Background:**

Formalin‐fixed paraffin‐embedded (FFPE) tissue is the standard material for companion diagnostic tests (CDx). Although cytological specimens can be useful, their low tumor content (TC) poses challenges. Recently, a high‐sensitivity CDx, the “Lung Cancer Compact Panel (cPANEL),” with a recommended TC of 5%, was introduced into clinical practice. This CDx achieves high detection sensitivity by selecting specific genes for analysis, processing them separately, and performing sequence at sufficient depth. This approach enables the detection of mutations even when they are present in only small amounts within the sample. This study aimed to evaluate the utility of cPANEL using FFPE cell block (CB) with a low TC.

**Methods:**

We analyzed FFPE‐CB pleural fluid samples from 26 patients diagnosed with malignant pleural effusion originating from lung adenocarcinoma. TC was quantified using AI‐equipped image analysis software, and samples with a TC of less than 10%—the minimum threshold for single‐CDx—were selected for CDx analysis. To confirm that cPANEL has better sensitivity than single‐CDx, which has high detection power, and that it is advantageous in low TC samples such as FFPE‐CB, we compared the two CDx assays based on the EGFR‐positive rate using samples that did not meet the TC criterion for single‐CDx.

**Results:**

Most FFPE‐CB had low TC levels, with only approximately 27% meeting the TC criteria for the widely used multi‐CDx. Furthermore, over 60% of the samples fell below the 10% threshold for a single‐CDx. Despite the low TC, EGFR mutations were partially detected, with cPANEL achieving a higher detection rate than that of single‐CDx (68.8% vs. 50.0%).

**Conclusions:**

Although cPANEL is classified as a multi‐CDx, it demonstrated superior sensitivity compared with that of single‐CDx. It proved effective even for FFPE‐CB samples with very low TC, making it a promising tool to enhance the use of cytological samples for CDx in the future.

## 1 Introduction

To date, various molecular‐targeted agents have been developed and introduced into clinical practice, primarily for the treatment of lung cancer [1, 2, 3]. In molecular‐targeted therapy, target molecules are typically confirmed using immunohistochemistry (IHC), polymerase chain reaction (PCR), and comprehensive genomic profiling (CGP) via next‐generation sequencing (NGS). Some of these tests are considered companion diagnostic tests (CDx). In particular, patients with non‐small cell lung cancer (NSCLC) frequently undergo CDx in clinical practice because of the remarkable advances in molecular‐targeted therapies [4, 5].

Cytological specimens have long been explored for their potential as a target for genomic testing. However, the current standard material for genomic testing is formalin‐fixed paraffin‐embedded (FFPE) specimens from pathological diagnoses [6]. Although FFPE is convenient for storage and use, poor nucleic acid quality often presents challenges [7]. Biopsy specimens face challenges not only in quality due to formalin fixation but also in quantity, as they are typically small and also used for pathological diagnosis. By contrast, cytological specimens, such as smears, are fixed with ethanol, which reduces the risk of nucleic acid fragmentation caused by formalin fixation [8, 9]. Additionally, although cell block (CB) and some liquid‐based cytology samples are fixed with formalin, the processing is faster compared to tissue samples, and the fixation conditions can be more easily controlled. As a result, the nucleic acid quality of these specimens is often sufficient [10, 11, 12]. These advantages have enabled the validation of cytological specimens as viable candidates for CDx. However, their use in CDx is not as widespread as initially expected [13].

In Japan, the focus has shifted from single‐CDx using IHC and PCR to multi‐CDx based on NGS. This shift may explain why cytological specimens are not widely used for CDx. The success rate of genomic tests correlates with the quality and yield of nucleic acids [14]. By contrast, the alteration detection rate depends on tumor content (TC) [15]. NGS, including CGP, requires macrodissection of the tumor region to enrich for TC [16]. However, this process is challenging for cytological samples because, compared to tissue samples, the areas where tumor cells and normal cells exist are not separated and are mixed together in a complex manner. In general, a higher TC is required for alteration detection in multi‐CDx than in single‐CDx, which is disadvantageous for cytological samples where TC enrichment is limited. Therefore, in multi‐CDx tests that require high TC values, cytological samples are likely considered unsuitable for analysis, and their use is perhaps avoided.

In November 2023, the “Lung Cancer Compact Panel Dx Multiplex Companion Diagnostics Test” (cPANEL) (DNA Chip Research Inc., Tokyo, Japan) was developed and approved for insurance coverage in Japan. Using both DNA and RNA, CDx can detect most driver alterations (EGFR, ALK, ROS1, MET, KRAS, BRAF, RET) that are amenable to molecular‐targeted therapy in lung cancer. This CDx which uses NGS technology achieves high detection sensitivity by limiting driver alterations of NSCLC for analysis, dividing them into modules, processing them separately, and performing sequence analysis at sufficient depth. In fact, The limit of detection of this CDx is reported to be 0.1%–0.54% (for EGFR mutation only), making it as sensitive as or more sensitive than conventional single‐CDx tests, according to existing instructions and reports [17, 18]. As multi‐CDx has become the standard, this high‐sensitivity multiplex test is expected to play an important role in the effective utilization of cytological specimens for CDx, especially those with low TC levels.

In this study, we focused on samples with low TC and evaluated the utility of cPANEL using FFPE‐CB by comparing its mutation detection rate with that of the highly sensitive single‐CDx. For validation, we selected EGFR mutations, which are the most frequently detected in Japanese lung cancer patients.

## 2 Material and Methods

### 2.1 Case Selection and Sample Preparation

In this study, we analyzed the pleural fluid FFPE‐CB samples of 26 patients diagnosed with malignant pleural effusion originating from lung adenocarcinoma between 2016 and 2022 at Hokkaido University Hospital. FFPE‐CB samples were prepared from pleural fluid at the same time as smear preparation for cytology. To prepare the FFPE‐CB, pleural fluid was first centrifuged at 3000 rpm for 3 min. The supernatant was discarded, and the cell pellet was fixed with 10% neutral‐buffered formalin (MUTO PURE CHEMICALS, Tokyo, Japan) for a specified time. The fixed cell pellets were dehydrated, permeabilized, and embedded in paraffin using a tissue processor (VIP6AI; Sakura Finetek Japan, Tokyo, Japan). The process of preparing FFPE‐CB in this study is the same as the one routinely used for pathological diagnosis.

### 2.2 TC Evaluation

The TC of FFPE‐CB was calculated using AI‐enabled Halo software (Indica Labs, Albuquerque, NM, USA). IHC preparations for TTF‐1 (clone: SP141, Roche Diagnostics, Basel, Switzerland), Ep‐CAM (clone: Ber EP4, Roche Diagnostics), and cytokeratin (clone: AE1/AE3, Roche Diagnostics) were used to assess TC, as these markers only stained tumor cells in pleural fluid specimens, allowing for more accurate tumor cell recognition by AI. The specimens used for IHC were prepared using the same method as that for pathological diagnosis (4‐μm sections were mounted on coated glass slides). Staining was performed using the VENTANA BenchMark ULTRA (Roche Diagnostics). After IHC preparations were captured as whole slide images using NanoZoomer 2.0HT (Hamamatsu Photonics, Shizuoka, Japan), TC was calculated by counting the total number of cells and the number of IHC‐positive cells on the whole slide images using Halo software. The TC cutoff was set at 10%, with cases exceeding this threshold defined as high‐TC and those below this threshold defined as low‐TC. Quantitative manual estimation of TC was not performed for direct comparison.

### 2.3 Nucleic Acid Extraction and Quality Control

Nucleic acids were extracted from FFPE‐CB using AllPrep DNA/RNA Kits (QIAGEN, Venlo, Netherlands) according to the manufacturer's instructions. Nucleic acid yield was measured by both absorbance (using a Nanodrop) and fluorescence (using a Qubit 2.0 Fluorometer; Thermo Fisher Scientific, Waltham, MA, USA). Nucleic acid quality was evaluated using a TapeStation (Agilent Technologies, Santa Clara, CA, USA). The DNA Integrity Number (DIN) and DV200 were used as metrics for the nucleic acid quality of the DNA and RNA, respectively, as these metrics correlate with the success rate of library preparation.

### 2.4 Companion Diagnostics of EGFR

**FIGURE 1:**
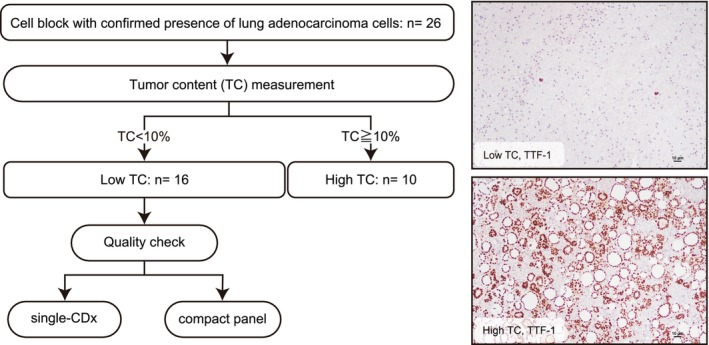
Flowchart depicting the analysis participants and procedures.

Both single‐CDx and cPANEL tests for EGFR detection were performed using nucleic acids extracted from FFPE‐CB in the case group classified as having low TC based on image analysis. For single‐CDx, either the cobas EGFR Mutation Test (Roche Diagnostics) or the therascreen EGFR RGQ PCR Kit (QIAGEN) was used. The EGFR detection results for both single‐CDx and cPANEL were compared (Figure 1). The results of CDx in clinical practice using specimens other than FFPE‐CB from the group classified as having low TC in this study are shown in Table S1.

### 2.5 Statistical Analysis

Experimental data are presented as the mean ± standard deviation. The Student's *t*‐test was used to compare data distributions between the two groups. To evaluate the test result agreement, the concordance rate, Cohen's kappa coefficient, and McNemar's test were calculated. Statistical significance was set at *p* < 0.05. All statistical analyses and plots were generated using R software (version 4.3.3).

## 3 Results

**FIGURE 2:**
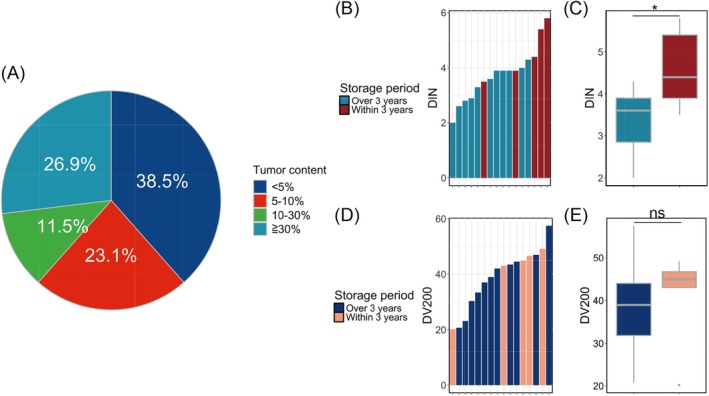
Sample conditions of cell block formalin‐fixed paraffin‐embedded (FFPE‐CB). (A) Pie chart showing the results of tumor content analysis. (B) DNA Integrity Number (DIN), which indicates DNA quality. Samples stored for more than 3 years and those stored for less than 3 years are displayed in different colors. (C) RNA quality, shown as the percentage of fragments longer than 200 nucleotides (DV200). The bar plot is colored according to the storage period. (D) DNA quality and (E) RNA quality by storage period of FFPE‐CB, displayed as boxplots. *p < 0.05; ns, not significant.

The total cell counts for the FFPE‐CB samples used in this study were 3.7 × 10^5^±2.7 × 10^5^ (max = 11.5 × 10^5^, min = 0.9 × 10^5^), tumor cell count was 7.9 × 10^4^±13.6 × 10^4^ (max = 58.8 × 10^4^, min = 0.007 × 10^4^), and TC was 16.4%±18.8% (max = 58.3%, min = 0.03%). The threshold for TC in some gene panel tests or CGP tests (including Oncomine CDx and FoundationOne CDx) is set at 30%, and only 26.9% of the cases (seven out of 26) exceeded this threshold. Additionally, only 38.4% of the cases (10 of 26) had TC values exceeding 10% (high‐TC group), which is the cutoff for most single‐CDx tests, whereas 61.6% of the cases (16 of 26) had TC values below 10% (low‐TC group) (Figure 2A). EGFR single‐CDx and cPANEL tests were performed on the low‐TC group (*n* = 16).

Figure 2B,C show that the nucleic acid quality of most FFPE‐CB samples used for CDx in this study met the minimum criteria. The DIN value was 3.76 ± 0.969, and the DV200 was 38.9% ± 10.7%. Only one case had a DIN value below 2.5, which is the threshold required for stable results [14]. Furthermore, all low‐DIN samples were FFPE samples stored for more than 3 years. Regarding RNA, while most RNA samples were of low quality, none fell below the recommended value (DV200 > 30%). Nucleic acid quality by sample storage period revealed that for DNA, the DIN value was 3.38 ± 0.72 for samples collected between 2016 and 2020, whereas it was 4.60 ± 0.98 for samples collected later, indicating that newer samples had better quality (*p* = 0.0137). A similar trend was observed for RNA, with DV200 values of 38.0% ± 10.7% for samples from 2016 to 2019 and 40.8% ± 11.7% for later samples. However, no significant differences in RNA quality were observed based on the timing of specimen collection (*p* = 0.6442) (Figure 2D,E).

**FIGURE 3:**
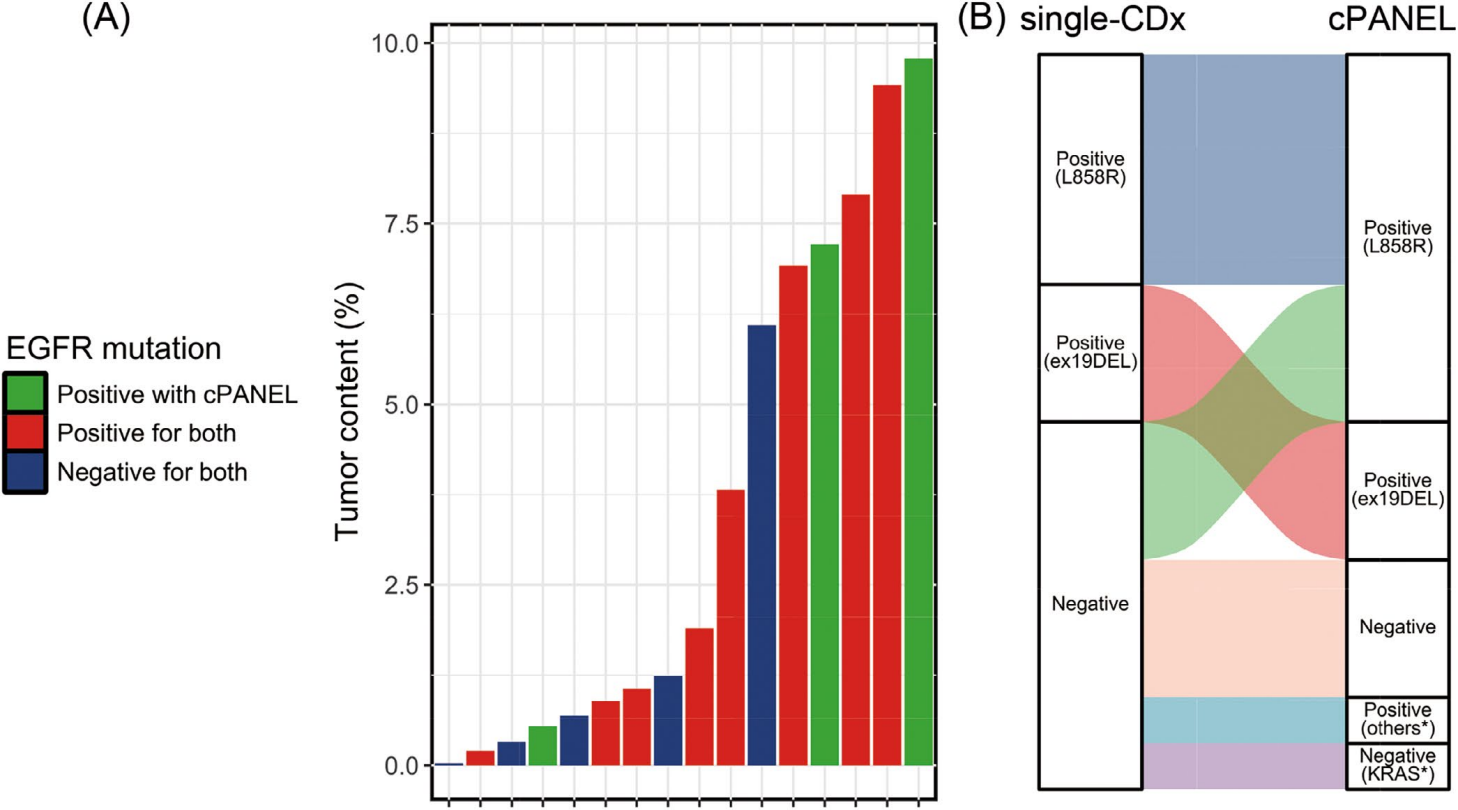
Comparison of the results between the Lung Cancer Compact Panel (cPANEL) and single‐companion diagnostic tests (Single‐CDx). (A) Tumor content in each sample, shown as a bar plot. Each bar is colored to indicate whether the results of cPANEL and Single‐CDx matched (positive or negative) or were positive in only one of the two tests. There were three cases where an EGFR mutation was detected only by cPANEL and not by Single‐CDx. (B) Alluvial plot of the detected variants in cPANEL and Single‐CDx. Asterisks in the plot indicate mutations and variants that are not included in the detection panel for Single‐CDx.

When comparing the positive rates of the two CDx tests for only the common EGFR variants, the positive rate of single‐CDx was 50.0% (eight of 16 cases), whereas the positive rate of cPANEL was 68.8% (11 of 16 cases); cPANEL detected EGFR positivity in the three cases that were negative for single‐CDx. There was no bias in the distribution of TC in FFPE‐CB samples from cases where the results did not match, and the variant allele frequency (VAF) detected by cPANEL was very low, approximately 1.0 (Figure 3A). Additionally, there were no discrepancies in the EGFR variants detected between single‐CDx and cPANEL in cases where both CDx were positive for mutations. The overall concordance rate between single‐CDx and cPANEL was 75.0% (12 of 16 cases), and Cohen's kappa coefficient was 0.50 (95% CI: 0.13–0.87), indicating moderate agreement. McNemar's test showed no statistically significant difference (*p* = 0.1336). Furthermore, although not directly related to this study, in addition to common variants of EGFR, cPANEL detected EGFR mutation variants that were not covered by single‐CDx and KRAS mutations in cases negative for single‐CDx (Figure 3B, Table 1).

**TABLE 1:** Single‐CDx and cPANEL results and parameter in TC‐low group.

	Single‐CDx		cPANEL		Cell block			
Case	Result	Method	Result	VAF (%)	Total cells	IHC positive cells	IHC negative cells	Tumor content (%)
2	Negative	therascreen	Negative	Negative	240,359	77	240,282	0.032
4	Negative	cobas	Negative	Negative	259,430	846	258,584	0.326
5	Exon21 L858R	cobas	Exon21 L858R	8	329,954	31,081	298,873	9.42
6	Exon21 L858R	therascreen	Exon21 L858R	92.5	351,532	27,791	323,741	7.906
7	Exon21 L858R	therascreen	Exon21 L858R	9.3	421,415	29,154	392,261	6.918
11	Negative	cobas	Exon21 V843I	1.2	438,848	5444	433,404	1.241
13	Negative	cobas	Exon21 L858R	1.8	432,702	42,365	390,337	9.791
14	Negative	cobas	Exon21 L858R	1.7	217,056	15,653	201,403	7.212
16	Exon21 L858R	cobas	Exon21 L858R	12	104,502	1107	103,395	1.059
17	Negative	cobas	Exon21 L858R	0.3	313,107	1695	311,412	0.541
18	Exon21 L858R	cobas	Exon21 L858R	32.1	133,560	2533	131,027	1.897
21	Exon19 del	cobas	Exon19 del	0.4	131,985	1178	130,807	0.893
22	Negative	cobas	KRAS G12V	5.3	524,507	31,957	492,550	6.093
23	Exon19 del	cobas	Exon19 del	0.6	116,931	239	116,692	0.204
25	Exon19 del	cobas	Exon19 del	5.4	322,134	12,309	309,825	3.821
26	Negative	cobas	Negative	Negative	277,907	1923	275,984	0.692

Abbreviations: CDx, Companion Diagnostics test; cobas, cobas EGFR Mutation Test v2; cPANEL, Lung cancer compact panel; IHC, Immunohistochemistry; therascreen, therascreen EGFR RGQ PCR Kit; VAF, variant allele frequency.

## 4 Discussion

In the present study, we investigated the utility of high‐sensitivity CDx using FFPE‐CB. To evaluate its utility, we focused on TC, mutation detection rates, and nucleic acid quality. Additionally, CDx is commonly performed, especially in lung cancer, and EGFR is the most prevalent driver mutation in lung cancer in Asia, including Japan [19]. Therefore, we validated the utility of cPANEL by examining the EGFR mutation detection rate in lung adenocarcinoma cells in pleural fluid.

FFPE‐CB is expected to have the lowest nucleic acid quality among cytological specimens, owing to the fixation conditions [20]. However, DNA from most samples met the QC threshold required for successful NGS analysis [14]. Some samples, however, fell slightly below these criteria because they included samples stored for several years. The RNA quality was generally low, even in samples stored for relatively short periods. This suggests that RNA is more prone to quality degradation than DNA during specimen handling. Nevertheless, all the samples were successfully tested for both DNA and RNA under these conditions. Although the quality of nucleic acids in FFPE deteriorates over time, as reported in many studies and guidelines [21], it is still possible to perform tests even after several years of storage. These results suggest that the nucleic acid quality in FFPE‐CB is suitable for genomic testing and that the success rate of genomic testing years later can be improved by carefully managing the fixation conditions during sample preparation, especially for RNA. Furthermore, cPANEL demonstrated robustness in analyzing samples with slightly compromised nucleic acid quality, supporting the clinical utility of FFPE‐CB specimens for genomic testing in clinical settings.

Pleural fluid specimens with high cellularity were used in this study. However, because pleural fluids are often inflamed and contaminated with inflammatory cells, histiocytes, and mesothelial cells, the TC was below the recommended threshold of 10% for a single‐CDx in many cases. Even in these samples, a single‐CDx was able to detect the mutations. However, cPANEL detected EGFR mutations in three cases that were negative by single‐CDx. Statistical comparison showed moderate agreement between the two assays. Although McNemar's test was not statistically significant, this likely reflects cPANEL's ability to detect additional low‐VAF alterations missed by single‐CDx, rather than equivalent performance. The VAF of the mutations detected in these three cases was very low, with one case being particularly low (VAF = 0.3%). This result is consistent with the detection threshold of 0.14%–0.54% for EGFR mutations described in the cPANEL instructions and reports [17]. In some cases, the detected VAF on cPANEL was lower than expected based on the measured TC. Possible explanations include overestimation of TC, differences in amplification efficiency between tumor and normal cells, and dilution of VAF due to the presence of polyploid cells [22]. Furthermore, although cPANEL recommends the use of cell pellets [17], this study demonstrated that FFPE‐CB is also suitable for testing. Unlike cell pellets, FFPE‐CB are samples in which nucleic acids are expected to have undergone fragmentation and chemical modification due to formalin fixation. These formalin‐induced alterations are known to reduce the success rate and accuracy of NGS analysis. However, this study showed that cPANEL can detect mutations from a small number of tumor cells in such samples. In clinical practice, FFPE samples are routinely used in pathological diagnosis and can be stored at room temperature, making them much easier to handle than unfixed cell pellets. In addition, cytological smear preparations retain whole cells on the slide, requiring three‐dimensional observation. In contrast, FFPE samples are sectioned, allowing for two‐dimensional observation and easier evaluation of TC compared to smears. These empirical findings are important for promoting the future use of cPANEL in cytological specimens.

It is well known that multiple driver alterations can exist in lung cancer, commonly mutually exclusive. Therefore, when selecting a treatment, it is often necessary to test multiple genes to identify driver alterations in lung cancer, especially in NSCLC. This is the main reason why multi‐CDx is currently the standard; however, it has stricter requirements for nucleic acid quantity and TC than single‐CDx. Furthermore, although this may be related to the TC requirement, it has been reported that the mutation detection rate is lower for multi‐CDx than for single‐CDx [23]. In this context, the emergence of multi‐CDx, which has higher detection capabilities than those of single‐CDx (Table 2) and covers most driver alterations in lung cancer, may improve the alteration detection rates in lung cancer and expand the use of cytological specimens in CDx. Furthermore, even low‐VAF mutations in actionable genes such as EGFR can potentially influence treatment decisions if confirmed as true positives. Recent studies have demonstrated that patients with low‐VAF EGFR mutations may still benefit from targeted therapies [24, 25]. Detecting low‐VAF mutations using highly sensitive assays like cPANEL is thus clinically meaningful, especially in samples with limited tumor cellularity.

**TABLE 2:** The limit of detection for EGFR in CDx published by each manufacturer.

EGFR variant	Cobas	Therascreen	Oncomine DxTT	cPANEL
Exon19 del (%)	1.39–2.53	0.14–16.87	4.4	0.14
Exon21 L858R (%)	3.96–5.32	5.94	5.3	0.2
Exon18 G719X (%)	2.46–5.56	5.08–10.30	—	0.1
Exon20 ins (%)	1.26–6.81	2.4–11.6	7.49	0.54

*Note:* This table is a summary of the limit of detection of variants that are relatively common, extracted from the package inserts of each test kit.

Abbreviations: CDx, Companion diagnostics; Cobas, cobas EGFR Mutation Test v2; cPANEL, Lung cancer compact panel; Oncomine DxTT, Oncomine Dx Target Test Multi CDx system; therascreen, therascreen EGFR RGQ PCR Kit.

However, microscopic evaluation of histopathological or cytological specimens is necessary because even the most highly sensitive tests have limitations. cPANEL was able to detect mutations in samples with very low TC, but in clinical practice, it is generally unacceptable to use samples that fall below the manufacturer's standard (TC ≥ 5%, or at least 2%) because of an increased risk of false negatives. We were surprised to find that the TC calculated by AI‐based image analysis in this cohort was substantially lower than what we had anticipated based on our general clinical experience with FFPE‐CB specimens. This is likely due to the inherently lower accuracy of manual tumor purity assessments [26, 27], particularly in cytological specimens, where tumor and nontumor cells are heterogeneously intermixed. Because of this complexity, we did not perform a quantitative manual estimation of TC in this study. These findings underscore the potential for overestimation in subjective assessment and support the value of objective, reproducible methods such as AI‐based quantification. However, using this method in clinical practice is difficult in many institutions. Therefore, we need to develop simpler methods or algorithms for calculating TC in specimens with complex cell arrangements to achieve more accurate CDx using FFPE‐CB.

The limitation of this study is that it was based on a small number of cases at a single institution, and the methods used to prepare FFPE‐CB were not sufficiently standardized. Thus, they may differ from the TC distribution and positive detection rates observed in the results of this study. In the future, multicenter studies incorporating diverse FFPE‐CB preparation in clinical practice and TC distributions will be essential to evaluate the performance of cPANEL under broader clinical conditions. Furthermore, increasing the number of cases will allow for the evaluation of cPANEL's performance in detecting non‐EGFR and rare driver alteration using FFPE‐CB specimens. These efforts are expected to further establish cPANEL as a reliable and versatile companion diagnostic tool applicable to a wide range of cytological specimens.

## 5 Conclusion

FFPE‐CB exhibited a lower TC than expected. However, high‐sensitivity CDx, such as cPANEL, can adequately test such specimens. However, even with high‐sensitivity CDx, there are detection limits. We hope that this study will expand the use of FFPE‐CB for CDx, contribute to patient outcomes, and provide a reminder to check for TC, even when using a high‐sensitivity panel.

## Author Contributions

**Yoshiki Shinomiya:** conceptualization (equal), data curation (equal), investigation (equal), visualization (equal), writing – original draft (equal), writing – review and editing (equal). **Yuko Ishida:** conceptualization (equal), data curation (equal), investigation (equal). **Kanako C. Hatanaka:** conceptualization (equal), data curation (equal), investigation (equal), supervision (equal), writing – review and editing (equal). **Mayumi Yamamoto:** investigation (equal). **Ayae Nange:** investigation (equal). **Asami Okumura:** investigation (equal). **Yumi Wada:** investigation (equal). **Mitsuharu Abiko:** data curation (equal). **Tomohiro Shimizu:** investigation (equal). **Ryoko Watanabe:** investigation (equal). **Yamato Hashimoto:** investigation (equal). **Yoshiharu Sato:** investigation (equal). **Jun Sakakibara‐Konishi:** supervision (equal), writing – review and editing (equal). **Tatsuya Kato:** supervision (equal), writing – review and editing (equal). **Shinya Tanaka:** supervision (equal), writing – review and editing (equal). **Yutaka Hatanaka:** conceptualization (equal), methodology (equal), project administration (equal), supervision (equal), writing – review and editing (equal).

## Ethics Statement

Approval of the research protocol by an Institutional Reviewer Board: The research protocol was approved by the Institutional Review Board of Hokkaido University Hospital (no. 021‐0063).

## Consent

All informed consent was obtained from the subjects and/or guardians.

## Conflicts of Interest

Kanako C. Hatanaka received honoraria from Eli Lilly and AstraZeneca, research funding from Sakura Finetek Japan and Sekisui Medical, and travel, accommodations, and expenses from Chugai Pharma and AstraZeneca. The author has served on the speakers' bureau for Chugai Pharma, Sakura Finetek Japan, and AstraZeneca. Yoshiharu Sato is an employee of DNA Chip Research Inc. Yutaka Hatanaka received honoraria from AstraZeneca, MSD Oncology, Daiichi Sankyo, Merck, and Eli Lilly, research funding from Eli Lilly, NEC Corporation, CURED, Konica Minolta REALM, and Daiichi Sankyo. The author also served on the speakers' bureau for AstraZeneca. The remaining authors declare no conflicts of interest.

## Supporting information


**Table S1.** Results of CDx using samples other than cell blocks in low‐TC group.

## Data Availability

All data analyzed during this study are included in this article. Further enquiries can be directed to the corresponding author.
