# Correction to “Utility of a Novel High‐Sensitivity Multiplex Companion Diagnostic Test Using Formalin‐Fixed Paraffin‐Embedded Cell Block Materials of Non‐Small Cell Lung Cancer”

**DOI:** 10.1002/cam4.71100

**Published:** 2025-07-29

Shinomiya Y, Ishida Y, Hatanaka K. C., et al. Utility of a Novel High‐Sensitivity Multiplex Companion Diagnostic Test Using Formalin‐Fixed Paraffin‐Embedded Cell Block Materials of Non‐Small Cell Lung Cancer. *Cancer Medicine*. 2025;14(13):e71028. doi: 10.1002/cam4.71028

In the published article, the author's name “Mayumi Yamamoto” was incorrectly listed. The correct name should be “Mamiko Yamamoto,” as shown in the author list and author contribution statement.

We apologize for this error.

