# Supplementary material for: Utility of a Novel High‐Sensitivity Multiplex Companion Diagnostic Test Using Formalin‐Fixed Paraffin‐Embedded Cell Block Materials of Non‐small Cell Lung Cancer
Source: Cancer Med. 2025 Jul 4;14(13):e71028. doi: 10.1002/cam4.71028 (PMC12227799; doi:10.1002/cam4.71028)
Supplement: Supplementary file 1 — Table S1. Results of CDx using samples other than cell blocks in low‐TC group. [file CAM4-14-e71028-s001.docx]

Table S1. Results of CDx using samples other than cell blocks in low-TC group.

| Case | Result | Material | CDx Method |
| --- | --- | --- | --- |
| **2** | Negative | Pleural effusion | therascreen |
| **4** | Exon19 del | Device wash | therascreen |
| **5** | Exon21 L858R | Surgical | unknown |
| **6** | Exon21 L858R | TBB | cobas |
| **7** | Exon21 L858R | TBB | cobas |
| **11** | Exon19 del | TBNA | therascreen |
| **13** | Exon21 L858R | Pleural effusion | cobas |
| **14** | Exon21 L858R | Surgical | therascreen |
| **16** | Exon21 L858R | TBB | cobas |
| **17** | Negative | Pleural effusion | cobas |
| **18** | Not tested | Not tested | Not tested |
| **21** | Exon19 del | TBB | cobas |
| **22** | Not tested | Not tested | Not tested |
| **23** | Exon19 del | TBNA | Oncomine |
| **25** | Exon19 del | Device wash | therascreen |
| **26** | Not tested | Not tested | Not tested |

Abbreviation: CDx, Companion Diagnostics test; cobas, cobas EGFR Mutation Test v2; therascreen, therascreen® EGFR RGQ PCR Kit; Oncomine, Oncomine Dx Target Test Multi CDx system; TBB, transbronchial biopsy; TBNA, transbronchial needle aspiration
